# Fifty-Sixth Annual Meeting of the New York Medical Society

**Published:** 1863-03

**Authors:** 


### FIFTY-SIXTH ANNUAL MEETING OF THE NEW YORK MEDICAL SOCIETY

The Society met pursuant to Statute, in the room of the Court of Appeals, Capitol, at 11 o'clock Tuesday morning, and was called to order by the President, Dr. Thomas Hun, of Albany.

Dr. Hun then read his Inaugural Address, as follows:

The By-Laws of the Society make it the duty of the President, at the opening of the session, to speak of the condition of the Medical Profession in the State, and to call attention to such matters as may require the action of the Society. At this time our thoughts naturally turn first of all to the War in which our people are engaged, and to its influence on our profession. The State of New York was suddenly called on to furnish six hundred Surgeons and Assistant-Surgeons for the volunteer regiments, and when we take into account how utterly unprepared we all were by education and experience for such service, it must be admitted that the response to the call was quite as satisfactory as could have been expected. The medical care of the camp and military hospital differs so greatly from the duties of the practitioner in civil life as to demand a special training and discipline; and yet with the versatility of talents characteristic of our countrymen the Surgeons selected have not failed in any marked degree in the beginning, and are now rapidly improving. They certainly have not failed in self-devotion or in courage, and when we read of Surgeons who a few weeks before were going on their peaceful grounds, performing their duties under heavy fire, and remaining with the wounded to share their captivity, we have good right to be proud of our brethren.

They have not been deficient in moral qualities, nor so much in professional skill, as in attention to those regulations by which the health of camps is preserved. There has been too much reliance on the medicine chest and on surgical instruments, and too little care of diet, pure air, cleanliness, and other hygienic conditions. This is the common defect of our profession, both in civil as well as in military practice, but it has been more obvious in the latter because of the larger scale on which its consequences are exhibited.

The transactions of our Society for the last few years bear witness to the interest felt by the members both in public and in private hygeia, a subject which is far from having received all the attention it merits. It is to be hoped that this movement, which has at the foundation a most important reform in medical practice, may be continued.

Our profession is now, I believe, in a more sound condition than at any previous time. The standard of education in our principal medical schools is higher, and pupils go forth much more thoroughly educated than a few years ago. The war, with all its evils, is exercising a salutary influence in the medical profession. The examinations for admission to the medical service of the army and navy set up a high standard of excellence, and as this service presents a most desirable career for young men a strong inducement is held out to students to strive to come up to it. The benefit of these examinations is thus made to reach not only those who are successful in their application, but also all those who, though they fail, yet have tried to prepare for them. Their influence on the medical colleges is also most excellent, for students wishing to prepare for difficult examinations, will go to those colleges which afford the most instruction, and not to those in which degrees are most easily obtained. They have already led to the establishment of a chair of hygienic and military surgery in some of our Colleges. This serves to illustrate the working of a principle which has been proposed as a basis of reform and improvement in our medical schools, which would consist in the separation of the duties of teaching and examination, so that students might be induced to give preference, not to those Colleges which offer diplomas on the easiest terms, but to those which afford the best opportunities for preparing for examinations.

The American Medical Association has not met for the last two years, on account of the war, and it will be a matter of consideration whether the Society will recommend that a meeting of such members as can assemble under present circumstances shall be held.

I have received from the President of the Massachusetts Medical Society a communication addressed to the State Medical Society, on the subject of "Abuses in the Ambulance Service in the U. S. Army," which I will submit to the Society. I believe, however, this subject has received the attention of the proper authorities, and that the abuses complained of have been corrected.

It only remains for me to welcome you to our city, and to thank you for the honor you have conferred in calling me to preside over your deliberations.

Drs. Blatchford, Saunders and S. D. Willard, were named as a Committee on Credentials.

Drs. Armsby, Deering and Bissell, were appointed a Committee on the President's Address.

Drs. Cobb and Potter were appointed a Committee to invite medical members of the Legislature to a seat.

Dr. L. A. Smith, a delegate from the New Jersey State Medical Society was introduced, and was warmly received.

Dr. Hunt, of Hartford, and Dr. Beckwith, delegates from the State Medical Society of Connecticut, were also introduced to the Society.

The Committee on Credentials presented the names of the following gentlemen, invited to seats as Honorary Members during the sessions:—Benj. D. Carpenter, of Long Island; Cyrus Ramsey, of N. Y.; William Oakes, of Madison Co., N. Y.; Hiram McNutt, Warrensburgh, Warren Co.; J. Flint, Fort Edward, Washington Co.; William Seward, South Worcester, Otsego Co.; A. Grow, Schuylerville, Saratoga Co.; Hiram Watkyns, Walpole, N. H.

Dr. Taylor, of the Onondaga Medical Society, presented an address delivered by Dr. Israel Parsons, President of the Onondaga Society, before said Society at its annual meeting, on the subject of "Diphtheria." Referred to Publication Committee. Dr. Guido Furman, of N. Y., presented several communications from the New York County Medical Society, consisting of a "Report of the Meteorological Committee of the State;" also a report of "Case of Procidentia Uteri, of fifteen years' duration, with extreme ulceration of the neck," by Dr. Isaac E. Taylor. Referred to Publication Committee.

Dr. B. P. Staats, Chairman of the Board of Censors of the Eastern District, reported that they had examined and recommended to the President of their Society, for diplomas, the gentlemen whose names follow: J. Dufendorf, Montgomery Co.; Louis Applegate, Herkimer Co.; Wm. W. Squire, W. T. Calland, G. A. Chosley, John H. Burland, and Joseph Leman, of Montreal, Canada. Dr. Joel Foster, Chairman of the Board of Censors of the Southern District, reported that they had examined and recommended to the President of the Society for diplomas, Dr. Coles.

Dr. Blatchford announced the death of Zenas Cary, a delegate to the Society, and Dr. B. was directed to prepare an obituary notice for publication in the Transactions. Dr. Joseph Bates announced the death of Dr. Robert G. Tracy, of Hudson, Columbia County, and was requested to prepare an obituary notice for publication in the Transactions. Dr. Raphael announced the death of Dr. John C. Cheesman, and was requested to prepare an obituary notice for publication in the Transactions. The death of Dr. John T. Sheldon, a former delegate, was announced, and Dr. Ondinot was announced to prepare an obituary.

Dr. Cyrus Ramsey, Registrar of Records and Statistics, City of New York, presented a paper on the statistics of some of the diseases of New York and London.

Dr. Henry S. Downs, of New York, read a paper entitled "Post-Pharyngeal Abscess." Dr. Thomas C. Finnell presented a Pathological specimen, and made some remarks. Dr. Julius Auebrack read a paper entitled "De Lunatico Inquirendo."

The President announced the following Committee on Nominations: 1st district, Oliver White; 2d district, P. Stewart; 3d district; Thomas W. Blatchford; 4th district, James Ferguson; 5th district, N. H. Deering; 6th district, C. M. Crandall; 7th district, William Taylor; 8th distsict, H. W. Dean.

Dr. Lewis A. Sayre, of New York, read a paper entitled "A Remarkable Case of Deception." Dr. E. S. Arnold, of New York, presented a paper entitled "On Medical Provision for Railroads," which was referred to the Publication Committee. Dr. S. Barrett read a paper entitled "A Case of Delirium Tremens, treated by large doses of Digitals." Dr. Augustus Willard presented for Dr. Baker, of Chenango, a paper entitled "A Case of Lithotomy." Dr. Oakes presented a Pathological specimen passed from the bladder of a man in Hamilton.

AFTERNOON SESSION.

Dr. C. F. Taylor read a paper entitled "Treatment of Potts' Disease of the Spine." Dr. F. Hyde read a paper entitled "Fractures of the Cranium." Dr. B. Raphael gave a brief synopsis of a paper entitled "On the Ligation of Arteries." Dr. Charles A. Lee read a very valuable paper entitled "On Foreign Military Hospitals." Dr. George Cook read a paper entitled "A Case of Insanity." Dr, Case read a brief sketch of a case of disease of the Uterus.

SECOND DAY—FORENOON.

Dr. J. G. Adams, from the Committee appointed to attend the Connecticut Medical Society, reported. Dr. Squibb reported that the United States Pharmacopœia is now complete, except the preface and index. Dr. S. D. Willard presented a report from Dr. J. G. Orton on Medical and Surgical Statistics. The Committee was continued another year.

Dr. Blatchford reported a case of hydrophobia. Dr. D. G. Thomas read a paper on Ovarian Dropsy.

Dr. Armsby announced the bequest of $500 by the late Dr. M. H. Cash.

Dr. C. A. Lee offered resolutions approving the appointment of a Commissioner of Lunacy, and moved a Committee to confer with the Legislative Committee.

Dr. Corliss reported a case of enlarged liver. Dr. S. D. Willard presented papers by Dr. Gilfillan, of Brooklyn, on Tracheotomy in Diphtheria, and by Dr. Johnson on Exsection of the Ankle Joint. Dr. Willard presented an additional list of Surgeons in the Army from New York. Dr. Gardner read a paper on Ovariotomy. Dr. Quackenbush read a paper on Pelvic Presentation. Dr. March read a paper on the wound of Garibaldi, and presented an instrument for the removal of balls. Dr. Enos read a paper on Plaster of Paris dressing in club foot.

SECOND DAY—AFTERNOON.

Dr. Saunders presented a communication from the Madison County Medical Society, being an address delivered before said Society, on Diphtheria, by Dr. Saunders.

Mr. Brinsmade, from the Committee appointed to draft a Sanitary Code for the State of New York, made a partial report, and asked for further time to complete their labors.

Dr. Lee, from the Committee appointed to compare the Code of Medical Ethics adopted by this Society in 1823, with that of the American Medical Association, which has also been adopted (1847,) and present a revised copy to the Society at its next annual meeting, reported that they had carefully compared the two above mentioned, and that they find no essential difference between them, except that, in the Code adopted by the American Medical Association, it is stated to be derogatory to the character of a physician to take out a patent for a surgical instrument. As this subject was fully discussed at the last meeting, the Committee do not enter into its discussion, but present the following resolution:

*Resolved*, That a committee of three be appointed by the President to bring this subject before the next meeting of the American Medical Association, with a view to the re-consideration of this article.

After a brief discussion the resolution was indefinitely postponed.

Dr. Brinsmade, from the Committee appointed to report on Epidemics in the 3d Congressional District, reported that he had been unable to collect from medical gentlemen residing in the district the necessary statistics, and he, therefore, reported on the subject with reference to his own city, Troy. Referred to Pub. Com.

Dr. Sayre gave a synopsis of a report to be prepared for the Transactions on Hip Disease. Dr. Adams presented a paper prepared by Dr. T. B. Gunning on the "Treatment of Fractures of the Lower Jaw, by a new method."

Dr. Swinburne read a paper on "Exsections." Before concluding he gave way for a motion to adjourn, the conclusion of his paper being made the special order for Thursday morning.

On Wednesday evening the President, Dr. Hun, delivered an eloquent, truthful, and instructive address, a copy of which was solicited for publication in the Society's transactions.

THIRD DAY.

Dr. C. W. Crandall offered the following:

*Whereas*, The present civil war has caused the hospitals of the District of Columbia to be filled with sick and wounded soldiers from this State proportionate to the number of volunteers sent out.

*And whereas*, Every safeguard possible should be thrown around those who have perilled their all for us; therefore,

*Resolved*, That the New York State Medical Society respectfully request of our Legislators and Executive to earnestly consider the propriety of appointing an agent to reside at Washington, who shall be a Physician and Surgeon, with clerical assistants, whose only business shall be to look after the interest and welfare of the sick and wounded of the State of New York.

The Secretary read a communication from the Medical Society of Massachusetts, upon the subject of the Ambulance Service of the United States Army, calling attention to the gross abuses existing therein, and requesting sister societies to take action in the matter.

A discussion ensued, participated in by several gentlemen who had been on different battle-fields, showing the necessity for action in the premises.

Drs. Ordronaux, Garrish and Corliss, were appointed such Committee.

The Committee on Nominations presented their report, which was accepted, and the gentlemen named therein were elected:

President, Daniel P. Bissell, of Utica; Vice President, Joel Foster, of New York; Secretary, Sylvester D. Willard; Treasurer, John V. P. Quackenbush.—*Medical Times.*

